# Frequency of *CYP2D6* Alleles Including Structural Variants in the United States

**DOI:** 10.3389/fphar.2018.00305

**Published:** 2018-04-05

**Authors:** Andria L. Del Tredici, Alka Malhotra, Matthew Dedek, Frank Espin, Dan Roach, Guang-dan Zhu, Joseph Voland, Tanya A. Moreno

**Affiliations:** Millennium Health, LLC, San Diego, CA, United States

**Keywords:** CYP2D6, pharmacogenetics, drug metabolism, copy number variation, cytochrome P450

## Abstract

The *CYP2D6* gene encodes an enzyme important in the metabolism of many commonly used medications. Variation in *CYP2D6* is associated with inter-individual differences in medication response, and genetic testing is used to optimize medication therapy. This report describes a retrospective study of *CYP2D6* allele frequencies in a large population of 104,509 de-identified patient samples across all regions of the United States (US). Thirty-seven unique *CYP2D6* alleles including structural variants were identified. A majority of these alleles had frequencies which matched published frequency data from smaller studies, while eight had no previously published frequencies. Importantly, *CYP2D6* structural variants were observed in 13.1% of individuals and accounted for 7% of the total variants observed. The majority of structural variants detected (73%) were decreased-function or no-function alleles. As such, structural variants were found in approximately one-third (30%) of *CYP2D6* poor metabolizers in this study. This is the first *CYP2D6* study to evaluate, with a consistent methodology, both structural variants and single copy alleles in a large US population, and the results suggest that structural variants have a substantial impact on CYP2D6 function.

## Introduction

**Figure 1:**
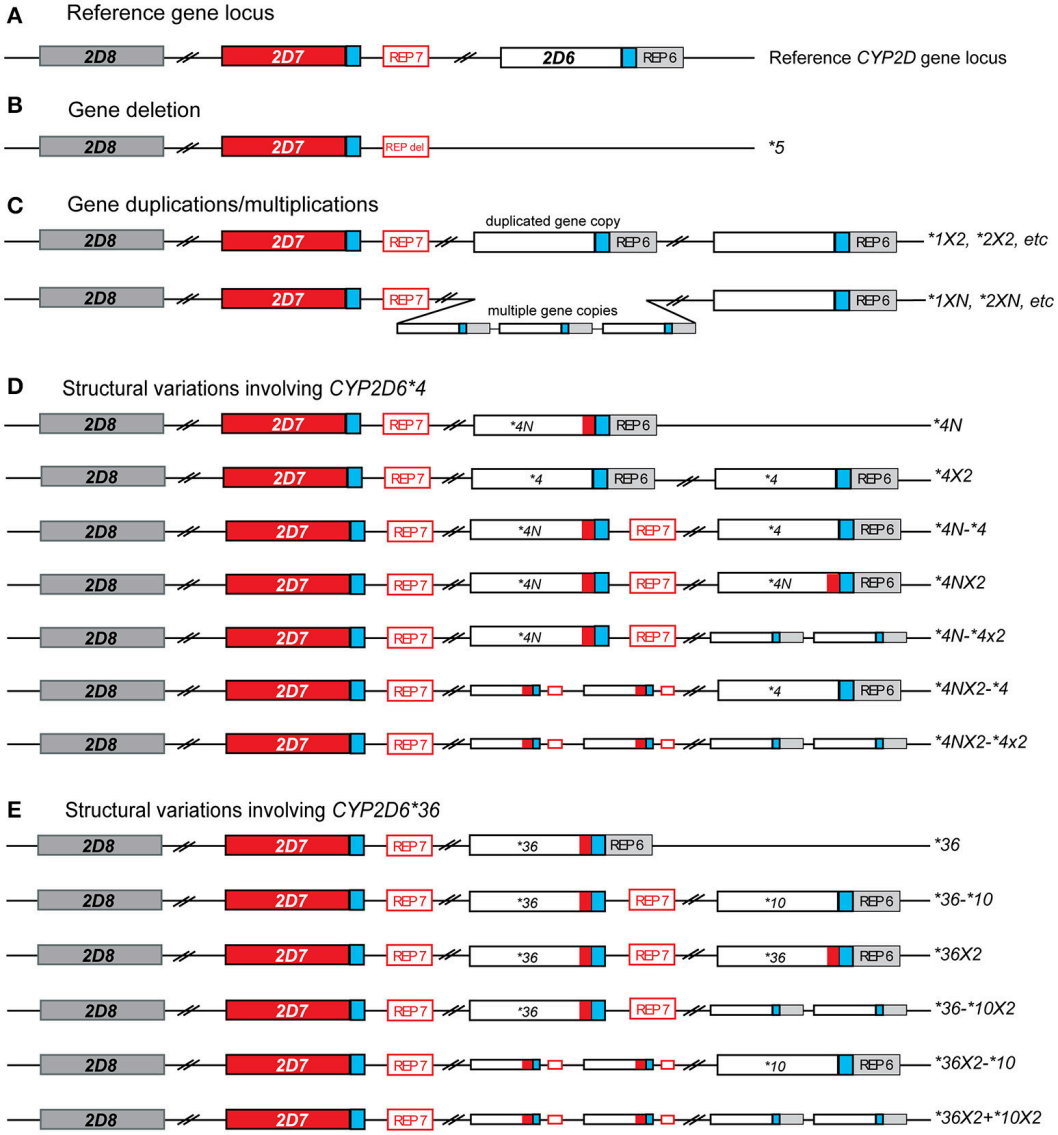
Overview of structural *CYP2D6* variants identified in this study. **(A)** Reference gene locus consisting of *CYP2D6* and two pseudo genes, *CYP2D7* (red), and *CYP2D8* (dark gray). Blue boxed indicate almost-identical downstream sequences and the open red and gray boxes labeled REP7 and REP6 represent regions with repetitive elements located downstream of *CYP2D6* and *CYP2D7*, respectively. **(B)** Deletion that includes the entire *CYP2D6* gene. The deletion breakpoints are within the near-identical REP6 and REP7 elements which are fused to the REP-DEL element in the CYP2D6*5 gene deletion allele. **(C)** Allelic variants carrying two or more identical gene copies. These may include duplications/multiplications of functional variants (i.e., *CYP2D6* *1XN) or no function variants (*CYP2D6* *4XN). This group does not include tandem arrangements, in which two or more different gene copies are present on an allele (see **E**). **(D)** Structural variants including *CYP2D6* *4. The red box within CYP2D6*4 gene copies indicates the presence of *CYP2D7*-derived sequences in exon 9, also known as the “exon 9 conversion” (these are designated CYP2D6*4N). **(E)** The *CYP2D6* *36 variant, which includes a *CYP2D7*-derived exon 9 conversion, can be found in different structural variants. The most commonly found is the *CYP2D6* *36-*10 tandem arrangement. In this study *CYP2D6* *36 alone was included in the single gene copy variants, not structural variants.

The CYP2D6 enzyme is involved in the hepatic metabolism of many clinically used medications (Zhou et al., 2009). The *CYP2D6* gene is highly polymorphic; over 100 allelic variants and subvariants have been designated to date by the Pharmacogene Variation Consortium (PharmVar) at www.PharmVar.org (Gaedigk et al., 2018). These include variations in single gene copies such as single nucleotide variants (SNVs) or insertions/deletions of a small number of nucleotides. The *CYP2D6* gene is also known to have structural variants, which include copy number variations (CNVs) such as the deletion of the entire *CYP2D6* gene, gene duplications and multiplications, as well as duplications/multiplications of non-identical gene units (also called tandems), and rearrangements involving the *CYP2D7* pseudogene (Figure 1). Consequently, the observed function of CYP2D6 is highly variable, ranging from poor (no enzyme activity) to ultrarapid (increased enzyme activity) metabolism (Owen et al., 2009). Patients with decreased or no CYP2D6 enzyme activity may be at risk of reduced efficacy and/or adverse effects when taking medications that are metabolized by the CYP2D6 enzyme. Guidelines for medication therapy adjustment based on *CYP2D6* genotype have been published for codeine, tricyclic antidepressants (TCAs), ondansetron and selective serotonin reuptake inhibitors (SSRIs) (Hicks et al., 2013, 2015; Crews et al., 2014; Bell et al., 2017) and are under review for tamoxifen.

*CYP2D6* allele frequencies have been evaluated in many ethnic groups, but the majority of studies have been relatively small (reviewed in Daly, 2015; Hicks et al., 2015; Gaedigk et al., 2017). More recently, a large meta-analysis of CYP2D6 allele frequencies has been published; while informative, one limitation is that data generated by numerous laboratories and methodologies was combined (Gaedigk et al., 2017). Another large study used NGS-based data from multiple sources, which is particularly hampered by technical limitations to quantify gene copy number variants from short read sequencing (Zhou et al., 2017). Indeed, discrepancies in *CYP2D6* test results from different laboratories have been observed and have been attributed to differences in assay design, alleles/variants tested, and allele nomenclature (Pratt et al., 2016). In addition, most studies have reported frequencies for allelic variants carrying a single gene copy, non-specific duplications and/or the CYP2D6^*^5 deletion, but did not further differentiate specific alleles in structural variants such as multiplication or tandem structures (Gaedigk et al., 2007; Yao et al., 2014; Beoris et al., 2016). To maximize accuracy of phenotype prediction from genotype data, it may be important to determine the nature of structural variants present in an individual (Gaedigk et al., 2007; Ramamoorthy and Skaar, 2011). The current study provides allele frequency information for *CYP2D6* genetic variants, including structural variants, using a consistent methodology in a large United States (US) population.

## Methods

A retrospective database analysis was conducted utilizing data from patients receiving *CYP2D6* pharmacogenetic testing from Millennium Health, LLC (San Diego, CA) between February 2015 and March 2016. Patients were excluded from analysis if: (1) no lab result was obtained due to insufficient quantity or poor quality of DNA or (2) the observed variations could not be ascribed to a known allele definition as described by the Pharmacogene Variation Consortium at www.PharmVar.org (Gaedigk et al., 2018). The latter was infrequently observed (<0.5%) and may have occurred when a patient had a rare allele that was not evaluated by the test, or carried a rare variant that interfered with an assay. The final analysis was performed on a population of 104,509 individuals, under a study protocol approved by Aspire IRB (Santee, CA).

*CYP2D6* pharmacogenetic testing was ordered by authorized healthcare providers using a requisition form in which patient ethnicity was selected from the following categories: African-American, Asian, Caucasian, Hispanic, or Other. Patients who did not belong to one of these ethnic groups or those with >1 ethnicity selected were identified as “Other.” Patients without ethnicity information were considered “Not Reported.” Data were available from all 50 states. Data from the US Census Bureau were used to categorize the population in terms of ethnicity, US geographic divisions, and genetic information (2016a; 2016b).

Choropleth maps were generated using a custom script in the R Statistical Programming language (version 3.1.1). For each region, the proportion of all individuals with each ethnicity category (African-American, Asian, Caucasian, Hispanic) was calculated. Patients who did not belong to one of these categories were excluded from the calculations. Choropleth maps were generated using the “ggplot2” R package (version 2.1.0). State boundaries were obtained from the “states” dataset of the “maps” R package (version 3.1.0) through the “map_data” function of the “ggplot2” package. State boundaries were merged into regions for display using the “sp” (SpatialPolygons) R package (version 1.2-3).

*CYP2D6* alleles are identified using an assay panel that simultaneously detects genotypes and CNVs of the gene. The panel is a clinical laboratory-developed test using TaqMan chemistry-based qPCR (Supplementary Table S1). The study laboratory is certified under the Clinical Laboratory Improvements Act (1988, CLIA), participates in *CYP2D6* proficiency testing and is accredited by the College of American Pathologists. External confirmation testing was performed at the Pharmacogenetics Core Laboratory, Children's Mercy Kansas City.

For *CYP2D6* testing, DNA was extracted from oral swabs (OCD-100, DNA Genotek, Ottawa, Ontario, Canada) or saliva collection devices (OGD-510, DNA Genotek, Ottawa, Ontario, Canada) using Chemagic DNA Saliva Kit (Perkin Elmer, Waltham, MA). The panel is comprised of assays targeting the allele-defining variants listed below (also see Supplementary Table S1; Gaedigk et al., 2018) as well as gene copy determination. For allele-defining variant detection, *CYP2D6* alleles were identified by specific allele-defining sequence variations as described by PharmVar; when no allele-defining sequence variations were identified, CYP2D6^*^1 was assigned as the wild-type (reference) allele (Gaedigk et al., 2018). The following *CYP2D6* alleles were included in this study with functional status as assigned: normal function: ^*^1, ^*^1xN, ^*^2, ^*^2xN, ^*^2A, ^*^2AxN, ^*^35, and ^*^35xN; decreased function: ^*^9, ^*^9xN, ^*^10, ^*^10xN, ^*^17, ^*^17xN, ^*^29, ^*^29xN, ^*^36-^*^10, ^*^36-^*^10xN, ^*^36xN-^*^10, ^*^36xN-^*^10xN, ^*^41, and ^*^41xN; and no function: ^*^3, ^*^3xN, ^*^4, ^*^4xN, ^*^4N, ^*^5, ^*^6, ^*^6xN, ^*^36, and ^*^36xN. The following CYP2D6^*^4-related structural variants were collapsed into one category in some figures as ^*^4xN: ^*^4N-^*^4, ^*^4N-^*^4xN, ^*^4NxN, ^*^4NxN-^*^4, ^*^4NxN-^*^4xN, and ^*^4xN. For structural variants that were gene multiplications, functional status was based on the status of the single gene copy.

Gene copy number was determined using two regions in the *CYP2D6* gene (intron 6 and exon 9) (Ramamoorthy et al., 2010). Samples were tested in 7 replicates and ΔΔCt was calculated to determine copy number for intron 6 and exon 9 with respect to two reference genes (*GAPDH* and *RNaseP*). Based on these calculations, samples were classified as having 0, 1, 2, 3 or >3 copies of *CYP2D6*. Alleles with 2 or more copies of *CYP2D6* were identified as “xN.” The CYP2D6^*^36 allele, which contains a *CYP2D7-*derived exon 9, was identified when the number of exon 9 copies was less than the number of intron 6 copies (Ramamoorthy et al., 2010). To assign copy number to the appropriate allele, samples were also analyzed for the relative proportion of wild-type vs. variant when variant alleles were present. Signal ratios detected by semi-quantitative qPCR for SNP detection in replicated assays were evaluated together with total copy number. For example, a sample with a copy number of 3, a wild-type:variant signal ratio of 2:1 for −1584C>G, and a wild-type:variant signal ratio of 1:2 for 1846G>A and 100C>T, would be assigned the genotype *CYP2D6* ^*^2A/^*^4xN (Supplementary Table S2). In another example, a sample with 1 copy of exon 9, 3 copies of intron 6, and a wild-type:variant signal ratio of 1:2 for 100C>T would be assigned the genotype *CYP2D6* ^*^1/^*^36xN.

For ambiguous genotypes, the most frequently observed genotype, according to published literature, was assigned. For example, a sample with a copy number of 2 and no allele-defining polymorphisms would be assigned CYP2D6^*^1/^*^1 rather than ^*^1xN/^*^5, since a CYP2D6^*^1/^*^1 genotype is more likely. Certain structural variants such as the CYP2D6^*^13-like alleles (in single or tandem arrangements) and ^*^68 were not specifically identified by this assay configuration. Samples carrying CYP2D6^*^13A-E alleles were likely called as CYP2D6^*^1 or ^*^2 instead of ^*^13. Samples containing the CYP2D6^*^68-^*^4 tandem were called as ^*^4.

Rarely, samples showed genotype, copy number, and/or signal data profiles that did not fit known allele profiles. In these cases, diplotypes were not called (<0.5%).

To assign predicted metabolizer status from genotype, these criteria were used (Owen et al., 2009): ultrarapid metabolizer (UM, ≥ 3 normal function gene copies); normal metabolizer (NM, 1 or 2 normal function alleles); intermediate metabolizer (IM, ≥ 2 decreased function alleles or 1 decreased function and 1 no function allele); poor metabolizer (PM, ≥2 no function alleles).

Frequency analysis was performed using Stata 14 software.

## Results

### Population

Data from 104,509 individuals were analyzed (Table 1). Patients ranged in age from 18 to 89 years. Physician-reported ethnicity information was available for 46,656 (44.6%) patients. More than 50% of patients did not have ethnicity information and 1,308 patients listed ethnicity as “Other.” Of the four ethnic groups, the Asian was the smallest with approximately 0.2% (*n* = 251) of all patients, and 0.5% of the patients with ethnicity information.

**Table 1:** Characteristics of study population.

	***N***	**%**
**GENDER^†^**		
Females	62,647	59.9
Males	41,527	39.7
**AGE**		
Mean	46.0 ± 16.1 years	
Range	18–89 years	
**ETHNICITY**		
African American	6,762	6.5
Asian	251	0.2
Caucasian	37,571	36
Hispanic	2,072	2
Other	1,308	1.3
Not reported^‡^	56,545	54.1
**Total**	104,509	100

† *335 patients did not have gender information*.

‡ *Not reported indicates that ethnicity information was not provided*.

**Figure 2:**
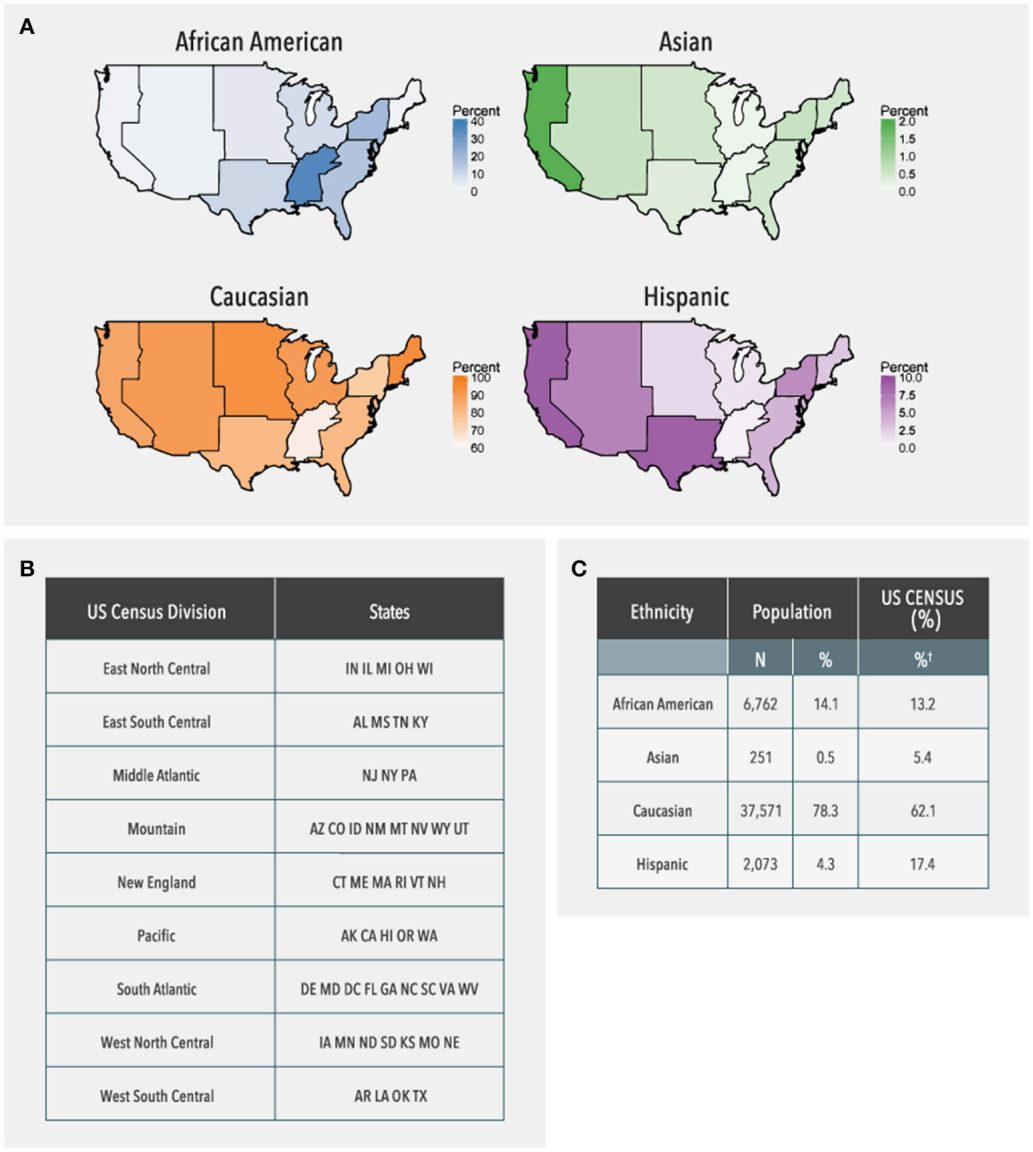
Ethnicity distribution of the study population based on geographical region**. (A)** US Maps shaded in proportion to the adjusted frequency of the study population for each ethnicity in each geographic region. The bar next to each choropleth map indicates how the shading varies based on frequency which is adjusted for the percentage of samples with reported ethnicity in each region (38–61%). Alaska and Hawaii are not shown on the choropleth, but were included in the data analysis. **(B)** Geographic regions as defined by states by the US Census Bureau. **(C)** Frequency of each ethnic group in the entire study population as compared to expected frequencies from the US Census Bureau. ^*†*^US Census data is provided only for those groups that are represented in the current study population; therefore, these percentages do not sum to 100%.

As shown in Figure 2, patient data were available from all US geographic regions. In the study population, Caucasians comprised the majority in all US geographic regions, with Hispanics more highly represented in Pacific, Mountain, and West South Central regions, and African Americans more highly represented in the East South Central and Middle Atlantic regions. Compared to the US Census data, Asians were under-represented (0.5% in the study population vs. 5.4% observed in the US Census data). Similarly, the proportion of Hispanics in the study population was lower compared to the US Census data (4.3 vs. 17.4%).

### Allele frequencies

All alleles detected were counted and calculated for their respective frequencies (Table 2). Thirty-seven distinct *CYP2D6* alleles were detected, including 23 structural variants. These included gene duplications (e.g., CYP2D6^*^1xN, ^*^2xN, ^*^4xN), tandem arrangements (e.g., CYP2D6^*^36-^*^10) and the CYP2D6^*^5 gene deletion. The most common no function allele was CYP2D6^*^4 followed by ^*^5, and the most common decreased function alleles were CYP2D6^*^17 and^*^41. Frequencies are reported for the first time in a large population for several structural variants: CYP2D6^*^2AxN, ^*^3xN, ^*^4N, some ^*^4N-type structural variants, ^*^9xN, and ^*^36xN-^*^10xN (Table 2). Out of these, ^*^2AxN had the highest frequency (0.9%).

**Table 2:** Frequency of alleles across the study population.

	**Allele**	***N***	**Obs freq %^†^**
**NO FUNCTION**			
	^*^3	2,854	1.4
	^*^3xN^‡^	**7**	**0**
	^*^4	33,588	16.1
	^*^4xN	1,140	0.6
	^*^4N^‡^	**99**	**0.05**
	^*^4N-^*^4	1,009	0.5
	^*^4N-^*^4xN^‡^	**15**	**0.01**
	^*^4NxN	7	0
	^*^4NxN-^*^4^‡^	**40**	**0.02**
	^*^4NxN-^*^4xN^‡^	**1**	**0**
	^*^5	7,066	3.4
	^*^6	2,076	1
	^*^6xN	15	0.01
	^*^36	241	0.1
	^*^36xN	95	0.05
**DECREASED FUNCTION**			
	^*^9	5,018	2.4
	^*^9xN^‡^^§^	**18**	**0.01**
	^*^10	3,637	1.7
	^*^10xN	168	0.1
	^*^17	5,674	2.7
	^*^17xN	49	0.02
	^*^29	2,926	1.4
	^*^29xN	46	0.02
	^*^36-^*^10	397	0.2
	^*^36-^*^10xN	8	0
	^*^36xN-^*^10	13	0.01
	^*^36xN-^*^10xN^‡^	**1**	**0**
	^*^41	17,085	8.2
	^*^41xN	66	0.03
**NORMAL FUNCTION**			
	^*^1	78,372	37.5
	^*^1xN	1,656	0.8
	^*^2	3,926	1.9
	^*^2xN	222	0.1
	^*^2A	30,052	14.4
	^*^2AxN^‡^	**1,781**	**0.9**
	^*^35	9,546	4.6
	^*^35xN	104	0.05

† *Numbers have been rounded to one decimal unless frequency ≤ 0.05*.

‡ *These bold-faced alleles do not have previously published frequency information*.

§ *One publication provides frequency information for this allele; however, the authors indicate this needs to confirmed using an independent method (Candiotti et al., 2005)*.

We characterized the frequency of *CYP2D6* alleles by ethnicity (Table 3). These frequencies agreed with published frequencies when available, as compiled by the Clinical Pharmacogenetics Implementation Consortium (Hicks et al., 2015). These frequencies also agreed with a more recent review of published allele frequencies (data not shown) (Gaedigk et al., 2017). For example, as expected, the frequencies of CYP2D6 ^*^17, ^*^29, ^*^4xN were highest in African-Americans compared to the other ethnic groups. Published frequencies, however, for all four ethnic groups were not available for all single copy alleles and structural variants detected (Table 3). Four of the single copy alleles and nine of the structural variants were missing published frequency data for at least 1 ethnic group.

**Table 3:** Frequency of alleles by ethnicity.

		**African American**				**Asian**				**Caucasian**				**Hispanic**			
	**Allele**	***N***	**Obs freq %^†^**	**Exp freq (Avg)^‡^**	**Exp freq (Min–Max)^‡^**	***N***	**Obs freq %^†^**	**Exp freq (Avg)^‡^**	**Exp freq (Min–Max)^‡^**	***N***	**Obs freq %^†^**	**Exp freq (Avg)^‡^**	**Exp freq (Min–Max)^‡^**	***N***	**Obs freq %^†^**	**Exp freq (Avg)^‡^**	**Exp freq (Min–Max)^‡^**
**(A) ALLELES WITH A SINGLE** ***CYP2D6*** **GENE COPY**																	
NO FUNCTION	^*^3	46	0.3	0.3	0–0.6	2	0.4^#^	0.01	0–0.03	1,188	1.6	1.3	0.5–2.2	33	0.8^#^	0.3	0–0.6
	^*^4	540	4.0	6.2	3.9–8	26	5.2	2.3	0–10.8	1,4062	18.7	19.4	17.5–21.0	527	12.7	15.9	10.0–20.6
	^*^4N	3	0.02	–^§^	–^§^	0	0	–^§^	–^§^	31	0.04	–^§^	–^§^	1	0.02	–^§^	–^§^
	^*^6	35	0.3	0.2	0–0.6	1	0.2	0.02	0–0.5	864	1.2	1.2	1–1.7	29	0.7^#^	0.2	0–0.4
	^*^36	62	0.5	0.6	0–1.0	22	4.4	1.5	0–16.4	12	0.02	0	0	1	0.2	–^§^	–^§^
	Total	686	5.1			51	10.2			1,6157	21.6			591	14.4		
DECREASED FUNCTION	^*^9	60	0.4	0.5	0–1.2	4	0.8	0.2	0–3.3	2,150	2.9	2.6	1.7–3.0	69	1.7	1.1	1.1
	^*^10	498	3.7	4.2	2.7–7.5	46	9.2	37.4	3.8–64.1	1,034	1.4	2.9	1.0–8.0	57	1.4	6.2	1.0–14.9
	^*^17	2,266	16.8	18.2	13.7–26.0	1	0.2	0.1	0–1.0	200	0.3	0.3	0.1–0.4	77	1.9	1.2	0.2–2.0
	^*^29	1,264	9.4^#^	6.5	5.0–7.7	0	0	0.02	0–0.2	71	0.1	0.1	0.02–0.1	66	1.6	0.2	0.2
	^*^41	338	2.5	9.4	1.8–14.9	22	4.4	3.1	0–12.5	7,175	9.6	8.5	7.6–9.8	238	5.7	5.5	5.5
	Total	4,426	32.7			73	14.5			1,0630	14.2			507	12.2		
NORMAL FUNCTION	^*^1	4,493	33.2	34.0	30.6–36.2	168	33.5	36.5	17.5–93.8	28,036	37.3	37.9	33.5–41.9	1,902	45.9	32.7	10.3–55.1
	^*^2	1,573	11.6	14.2	4.2–28.7	1	0.2^#^	16.7	7.7–42.7	192	0.3^#^	25.4	15.9–33.7	53	1.3^#^	33.8	22.0–45.6
	^*^2A	689	5.1	–^§^	–^§^	55	11.0	–^§^	–^§^	11,717	15.6	–^§^	–^§^	708	17.1	15.4	10.7–20
	^*^35	120	0.9	0.8	0.4–1.1	5	1.0^#^	0.1	0–0.9	4,189	5.6	4.8	4.8	123	3.0	–^§^	–^§^
	Total	6,875	50.8			229	45.6			4,4132	58.7			2,786	67.2		
**(B) STRUCTURAL VARIANTS**																	
NO FUNCTION	^*^3xN	**0**	**0**	**–**^§^	**–**^§^	**0**	**0**	**–**^§^	**–**^§^	**5**	**0.01**	**–**^§^	**–**^§^	**2**	**0.05**	**–**^§^	**–**^§^
	^*^4xN	387	2.8	2.1	0.3–4.1	1	0.2	0.03	0–0.3	622	0.8	0.3	0.1–0.4	21	0.5	0.2	0.2
	^*^5	710	5.3	6.1	2.8–8.7	14	2.8	4.9	0–15.8	2,332	3.1	2.5	0–3.8	118	2.9	2.8	1.7–4.6
	^*^6xN	0	0	0.2	0–0.7	0	0	0	0	3	0	0	0	0	0	0	0
	^*^36xN	1	0.01	0	0	27	5.4^#^	0.3	0–0.7	1	0	0	0	**2**	**0.05**	**–**^§^	**–**^§^
	Total	1,098	8.3			42	8.4			2,963	3.9			141	3.5		
DECREASED FUNCTION	^*^9xN	**0**	**0**	**–**^§^	**–**^§^	**0**	**0**	**–**^§^	**–**^§^	**6**	**0.01**	**–**^§^	**–**^§^	**0**	**0**	**–**^§^	**–**^§^
	^*^10xN	8	0.1	0.1	0–0.6	42	8.4^#^	0.3	0–1.0	11	0.01	0	0	2	0.05	0	0
	^*^17xN	25	0.2	0.2	0–0.7	0	0	0	0	0	0	0	0	0	0	0	0
	^*^29xN	18	0.1	0.2	0–0.7	0	0	0	0	3	0	0	0	**1**	**0.02**	**–**^§^	**–**^§^
	^*^36-^*^10	21	0.2	0.3	0.3	52	10.4^#^	26.4	22.5–32.7	43	0.1	0	0	**10**	**0.2**	**–**^§^	**–**^§^
	^*^36-^*^10xN	**1**	**0.01**	**–**^§^	**–**^§^	1	0.2	0.1	0–0.2	**1**	**0**	**–**^§^	**–**^§^	**0**	**0**	**–**^§^	**–**^§^
	^*^36xN-^*^10	**0**	**0**	**–**^§^	**–**^§^	2	0.4	0.8	0–1.3	**2**	**0.0**	**–**^§^	**–**^§^	**0**	**0**	**–**^§^	**–**^§^
	^*^41xN	3	0.02	0.3	0–1.2	0	0	0.03	0–0.2	18	0.02	0.1	0–0.1	0	0	0	0
	Total	76	0.6			97	19.3			84	0.1			13	0.3		
NORMAL FUNCTION	^*^1xN	109	0.8	0.4	0–1.2	5	1	0.3	0–0.6	602	0.8	0.3	0–0.7	32	0.8	0	0
	^*^2xN	21	0.2	1.6	0.2–2.4	0	0	0.5	0–1.6	75	0.1^#^	0.6	0.5–0.8	2	0.05^#^	0.6	0.6
	^*^2AxN	**232**	**1.7**	**–**^§^	**–**^§^	**5**	**1**	**–**^§^	**–**^§^	**460**	**0.6**	**–**^§^	**–**^§^	**65**	**1.6**	**–**^§^	**–**^§^
	^*^35xN	1	0.01	0	0	0	0	0	0	37	0.05	0.1	0–0.2	**7**	**0.2**	**–**^§^	**–**^§^
	Total	363	2.7			10	2			1,174	1.6			106	2.6		

† *Observed frequencies (Obs Freq) calculated as the proportion out of the total number of alleles (structural variants and alleles with a single copy combined)*.

‡ *Expected frequencies (Exp Freq) from CPIC^6^ (unless otherwise specified). While African American and Caucasian expected frequencies were identified from US cohorts, expected frequencies in Asians were selected from Chinese, Japanese, and Indian populations, and Hispanic expected frequencies were identified from Mexican American populations. Numbers have been rounded to one decimal unless frequency ≤ 0.05*.

§ *No published frequency data were available*.

# *Expected frequency is not within range of published literature*.

*CNVs, copy number variations. N denotes 2 or more gene copies*.

Some alleles had observed frequencies that were different from published frequencies (Hicks et al., 2015). CYP2D6^*^2 frequencies were lower than expected in Asians, Caucasians and Hispanics (Table 3A). In addition, the sum of observed CYP2D6^*^2 and^*^2A frequencies was similar to the published frequency of *CYP2D6* ^*^2. The same trend was observed for the CYP2D6^*^2xN and ^*^2AxN gene duplications (Table 3B). We also observed a lower frequency (10.4%) for CYP2D6^*^36-^*^10 in Asians compared to expected (22.5–32.7%) (Hicks et al., 2015). In contrast, CYP2D6^*^10xN and ^*^36xN were observed at a much higher frequency than expected in Asians (Table 3B).

When we evaluated allele frequencies for regional differences (data not shown), we found that East South Central had a higher observed frequency of CYP2D6^*^17 (2.3%) and CYP2D6^*^29 (2.4%). These alleles are known to be found predominantly in individuals with African ancestry (Hicks et al., 2015), consistent with the higher proportion of African-Americans in this region (Figure 2). Similarly, the West North Central region which had the highest proportion of Caucasians also had higher frequencies of CYP2D6^*^4 (13.6%) and CYP2D6^*^41 (6.9%) which are more common in Caucasians (Hicks et al., 2015).

**Figure 3:**
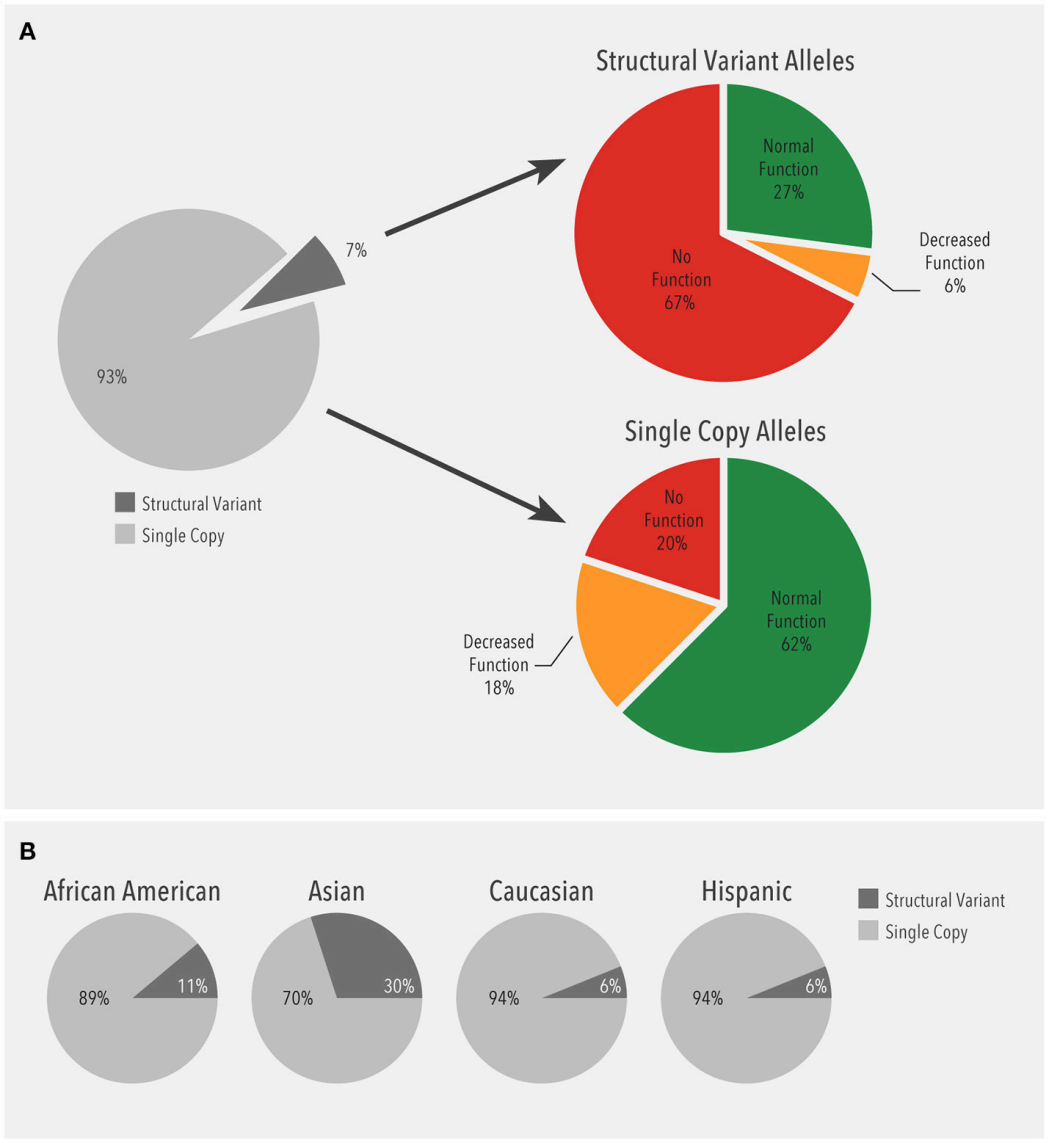
**(A)** Frequency of alleles with a single gene copy (*n* = 195,094) and structural variants (*n* = 13,924). Structural variants (SV) include copy number variants (CNVs) such as gene deletions and duplications, tandems, and rearrangements. **(B)** Ethnicity-specific frequency of alleles with a single gene copy and structural variants. Structural variants (SV) include copy number variants (CNVs) such as gene deletions and duplications, tandems, and rearrangements.

Next, the distribution of alleles carrying a single gene copy and alleles with structural variants were compared. Structural variants were observed in 13,693 (13.1%) patients. Of all alleles detected (209,018), 93% (195,094) were single copy alleles while 7% (13,924) were structural variants (Figure 3A). Among the single copy alleles, the majority were normal function (62%). In contrast, the majority of the structural variants had no function (68%), which was largely due to the CYP2D6^*^5 gene deletion (51%). As such, duplications of no function alleles (*n* = 2,329) accounted for 17% of no function structural variants. Moreover, the proportion of structural variants with no or decreased function (72%) was much higher than the corresponding proportion of single copy variants (38%).

When the distribution of structural variants was analyzed by ethnicity, single copy alleles accounted for the majority (89–94%) of alleles (Figure 3B). Interestingly, the proportion of structural variants was higher in Asians (30%) compared to the other ethnicities (6–11%).

**Figure 4:**
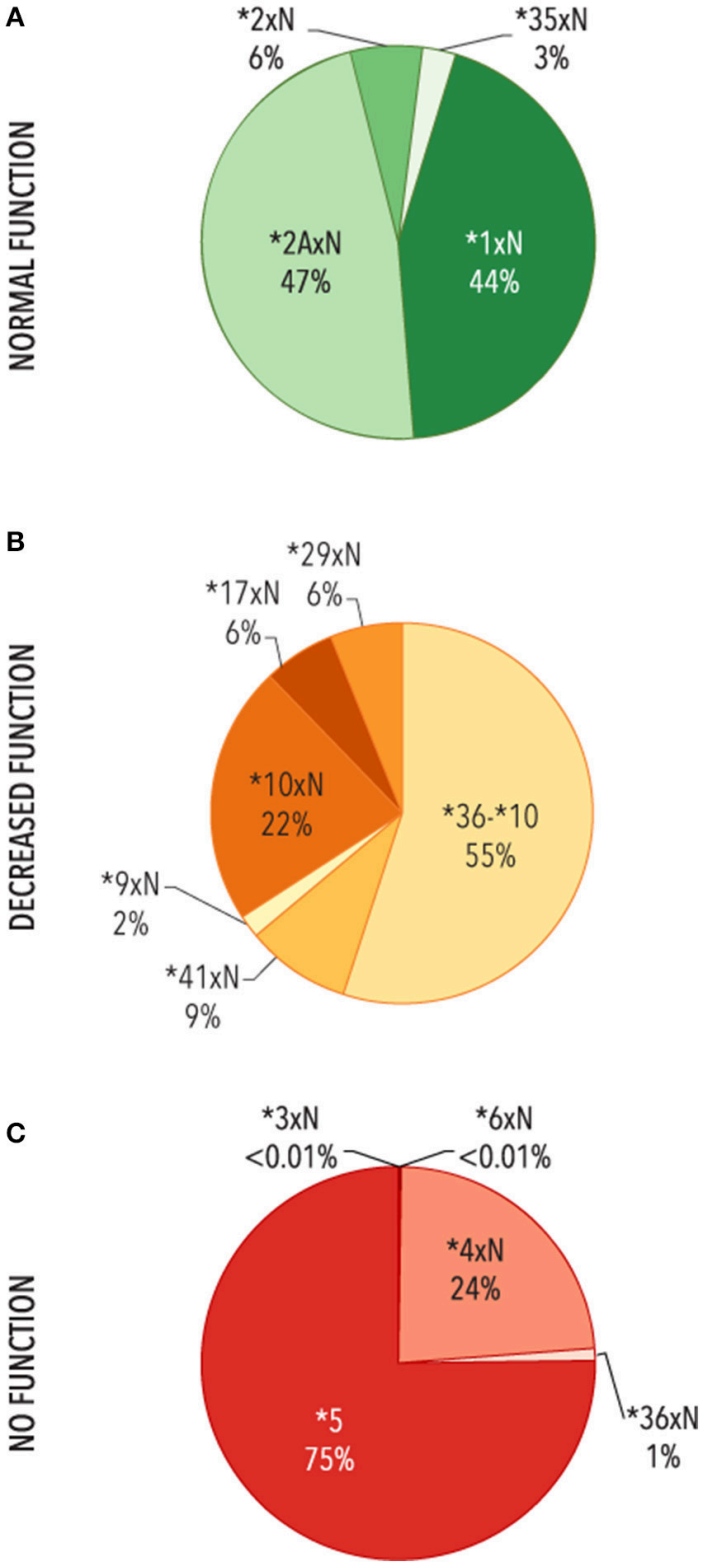
Functional categories of structural variants. Frequency of alleles which include **(A)** 2 or more copies of normal function genes (*N* = 3,763)**, (B)** ≥ two copies of decreased function variants or tandems with decreased function (*n* = 753), **(C)** gene deletions (*CYP2D6* *5) or ≥ 2 copies of variants with no function (*n* = 9,395). Functional status is defined based on the functional category for the single gene copy. *4xN represents *4N-*4, *4N-*4xN, *4NxN, *4NxN-*4, *4NxN-*4xN, and *4xN. 36-*10 represents *36-*10, *36-*10xN, *36xN-*10, and *36xN-*10xN.

The contribution of each structural variant was then evaluated (Figure 4). The most common variants with two or more functional gene copies were CYP2D6^*^1xN and ^*^2AxN (44 and 47%, respectively). Of the structural variants with two or more decreased function gene copies, CYP2D6^*^36-^*^10 made up the majority (51%) followed by CYP2D6^*^10xN (24%) with the remaining 25% comprised of CYP2D6^*^9xN, ^*^17xN, ^*^29xN, ^*^36xN-^*^10, and ^*^41xN. The CYP2D6^*^5 gene deletion was the most common structural variant with no function (75%) followed by ^*^4xN and ^*^4xN-like variants (24%).

### Phenotype prediction from genotype data

**Figure 5:**
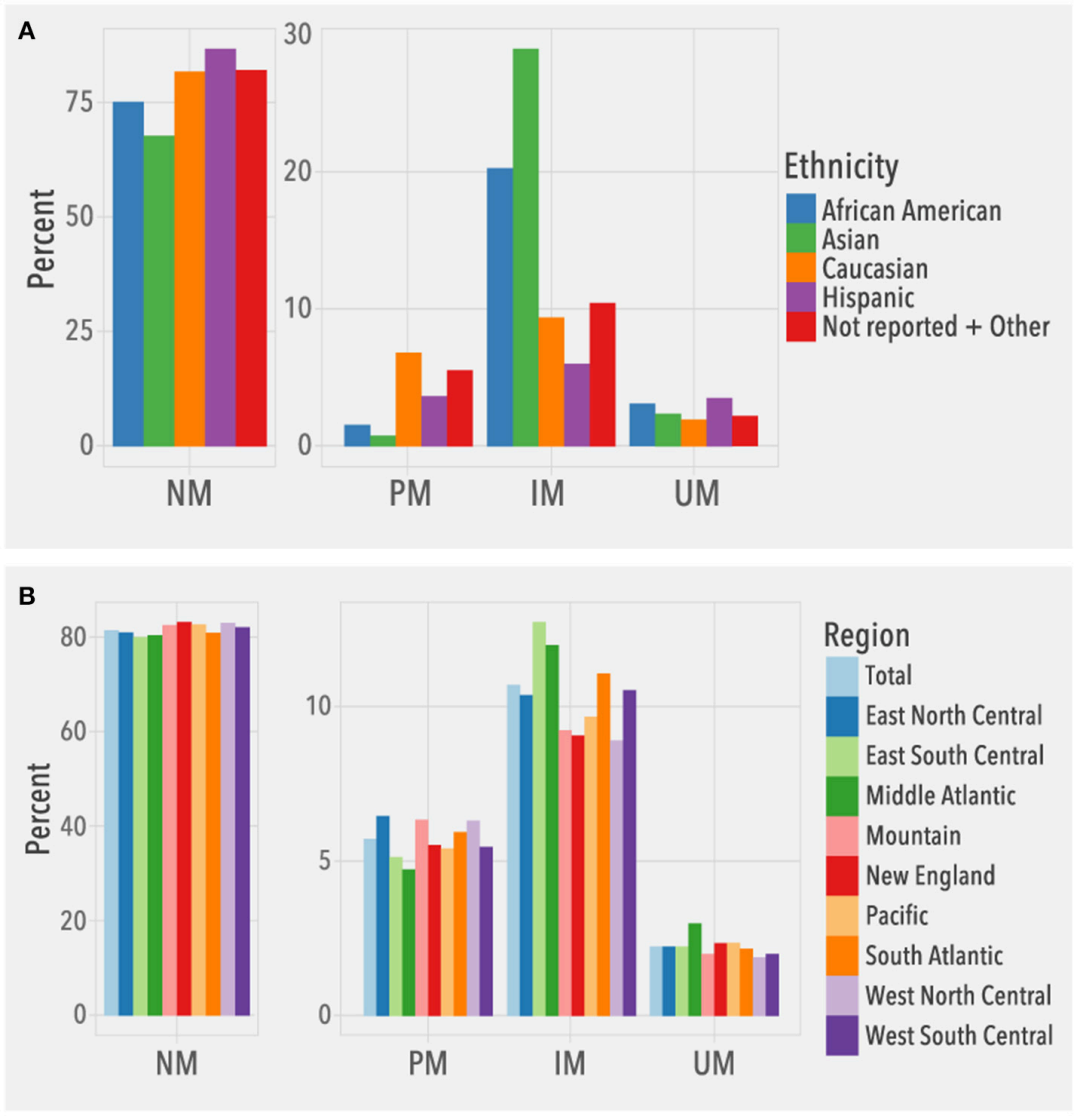
Predicted phenotype frequencies. Frequencies are based on **(A)** ethnicity (*N* = 104,509) and **(B)** geographical regions (*N* = 104,384; Puerto Rico not included). NM, normal metabolizer, PM, poor metabolizer, IM, intermediate metabolizer, UM, ultrarapid metabolizer.

To understand the clinical relevance of the observed *CYP2D6* allele frequencies, we evaluated the frequencies of predicted phenotypes. Among the 104,509 patients, 2,329 (2.2%) were predicted to be ultrarapid metabolizers (UMs), 85,021 (81.4%) normal metabolizers (NMs), 11,172 (10.7%) intermediate metabolizers (IMs), and 5,987 (5.7%) poor metabolizers (PMs). Overall, predicted phenotype frequency distributions (Figure 5A) were consistent with published data (Bernard et al., 2006; Gaedigk et al., 2017). For example, IMs were highest in Asians, and among Caucasians, the number of PMs was higher than UMs. The different US regions had similar distributions of predicted phenotypes based on the known ethnic diversity of each region (Figure 5B).

**Figure 6:**
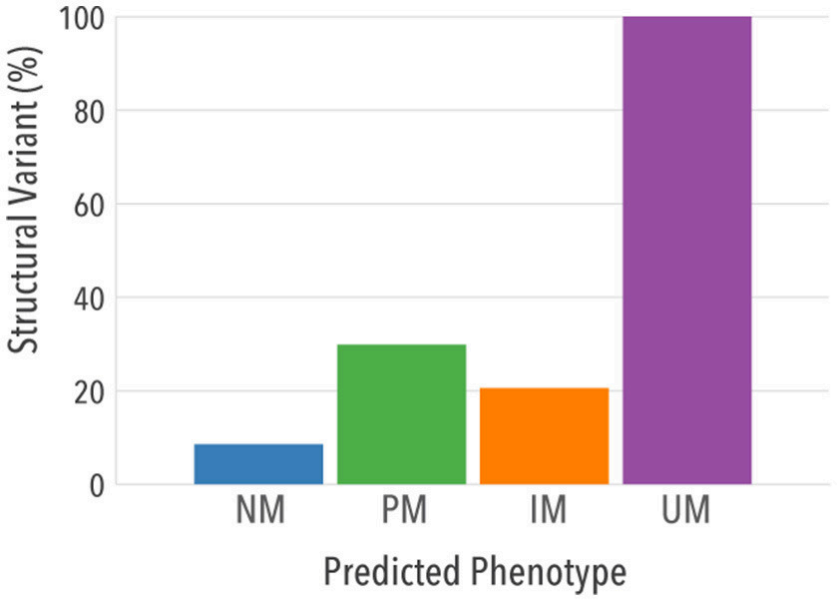
Contribution of structural variants and single copy variants to predicted phenotypes. Proportion of individuals in each predicted phenotype that had at least one structural variant is shown.

When structural variants were analyzed separately, 100% UMs had at least one allele carrying a duplication/multiplication of a normal function allele, as expected per definition (Figure 6). Importantly, a higher proportion of structural variation was found in PMs [*n* = 1,787 (29.8%)] and IMs [*n* = 2,294 (20.5%)] compared to NMs [*n* = 7,283 (8.5%)], indicating that structural variants may contribute substantially to reduced CYP2D6 activity *in vivo*.

## Discussion

In this retrospective observational study, *CYP2D6* allele frequencies were calculated from clinical data for individuals from multiple ethnic groups in all regions in the US. In particular, we found that structural variants have both substantial frequencies and potential functional impact. These results suggest that *CYP2D6* genetic tests that are able to detect a broad range of structural variants may be more predictive for CYP2D6 phenotype compared to tests that do not.

In this study, frequency information was observed for the first time for several alleles: CYP2D6^*^2A, ^*^2AxN, ^*^3xN, ^*^4N, and ^*^4N-like structural variants, ^*^9xN, and ^*^36xN-^*^10xN. In addition, some alleles were detected for the first time in certain ethnicities: ^*^29xN, ^*^35xN, ^*^36xN, ^*^36-10 in Hispanics, ^*^36-10xN, ^*^36xN-^*^10 in Caucasians, and ^*^36-10xN in African-Americans. Notably, most of the observed structural variants confer decreased or no enzyme function. We also found that 13.1% of subjects carried CNVs, similar to the 12.6% found in a smaller US study describing *CYP2D6* CNVs (*n* = 31,563) (Beoris et al., 2016). This study, however, did not further characterize gene duplications/multiplications as to the specific variant that was duplicated (i.e., did not determine whether a CYP2D6^*^1xN, ^*^4xN, etc. was present); in addition, the CNV assay only targeted the *CYP2D6* exon 9 region, an approach that does not adequately capture CNVs, and therefore may misclassify some tandem arrangements as “duplications” while missing others (Ramamoorthy et al., 2010; Ramamoorthy and Skaar, 2011; Gaedigk et al., 2012). Thus, while we replicated the finding that CYP2D6^*^5 is the most commonly found structural variant with no function, we also found that CYP2D6^*^4xN accounted for almost 25% of structural variants with no function. In addition, we also found that 5% of structural variants had decreased function, with *CYP2D6* ^*^36-^*^10 and ^*^10xN accounting for 3/4 of the alleles in this functional group.

The frequency data from this study was generated from a single laboratory accredited for pharmacogenetic testing. In contrast, other recently published studies combine data from multiple studies and/or laboratories (Hicks et al., 2015; Gaedigk et al., 2017; Zhou et al., 2017). Laboratories can differ in test design, including what alleles are interrogated and how alleles are identified, and these differences can lead to inconsistencies in results for the same sample (Pratt et al., 2016). In general, there is less published data on structural variants compared to single copy variants. For example, a recent sequencing study of more than 60,000 individuals included most major *CYP2D6* single gene copy variants but frequency information for structural variants were obtained from the literature due to technical limitations (Zhou et al., 2017).

Nonetheless, when published frequencies were available, a majority of observed allele frequencies were in agreement. However, some alleles had notable discrepancies, which may be explained by differences in testing technology and/or allele assignment. For example, the CYP2D6^*^2 allele was found at a frequency lower than expected, but the combined frequency of CYP2D6^*^2 and ^*^2A was comparable to published frequencies for CYP2D6^*^2. These observations are consistent with differences in testing and/or assignment of CYP2D6^*^2, which can vary between laboratories (Kalman et al., 2016). While our laboratory defines the CYP2D6^*^2 allele as having 2850C>T and ^*^2A as also having−1584C>G (Supplemental Table S1; Gaedigk et al., 2018), other laboratories do not distinguish the CYP2D6^*^2A subvariant and simply assign CYP2D6^*^2. Indeed, published frequency information on CYP2D6^*^2A is restricted to two small studies in Mexican and Ashkenazi Jewish populations (Scott et al., 2007; Sosa-Macías et al., 2010). There is emerging evidence that not all CYP2D6^*^2 subvariants convey the same level of activity (Wang et al., 2014, 2015). Thus, a testing strategy that includes such subvariants may be preferred so that clinical interpretations can be updated as subvariant functional studies are published.

Most structural variants also had similar frequencies to expected, when published frequencies were available. However, there was a notable discrepancy for CYP2D6^*^36xN and ^*^36-^*^10. These alleles had observed frequencies in Asians of 5.4 and 10.4%, respectively, in contrast to previously published studies of <500 individuals where CYP2D6^*^36xN was lower (0–0.6%) and CYP2D6^*^36-^*^10 was observed at a higher frequency (24.2–26.3%) (Gaedigk et al., 2006; Hosono et al., 2009; Kiyotani et al., 2010). These differences may be explained by differences in test panels. If a test does not identify the presence of the *CYP2D7*-derived exon 9 conversion, CYP2D6^*^36 would be misclassified as ^*^10 (Ramamoorthy et al., 2010; Ramamoorthy and Skaar, 2011; Gaedigk et al., 2012). Such a misclassification may have clinical implications since CYP2D6^*^36 is a no function allele, while ^*^10 is classified as decreased function. Similarly, the observed frequency of CYP2D6^*^10xN was higher than expected, suggesting that a fraction of CYP2D6^*^10xN duplications/multiplications were missed and reported as CYP2D6^*^10 in studies that lack CNV testing. Accordingly, frequencies for CYP2D6^*^10 and structural variants containing CYP2D6^*^10 vary substantially and depend on the capability to detect specific structural variation.

Indeed, we found that structural variants may play a major role for accurate prediction of CYP2D6 enzyme activity. Unlike single copy variants, in which normal function variants dominate, more structural variants were found with decreased and no function compared to normal function. This is likely due to the higher frequency of the gene deletion *CYP2D6* ^*^5 (3.4%) compared to duplications/multiplications of normal function alleles (combined frequency of 1.85% for *CYP2D6* ^*^1xN, CYP2D6 ^*^2xN, CYP2D6 ^*^35xN). Consistent with this observation, structural variants were found in more IMs and PMs compared to NMs. These results suggest that individuals carrying certain structural variants could be misclassified by a *CYP2D6* test that did not specifically identify these. Perhaps a broad panel of alleles, including structural variants, should be tested to ensure accurate *CYP2D6* genotype and therefore phenotype prediction.

We recognize some limitations of our study. We cannot rule out a selection bias since this study population consists of patients selected by their physicians to receive *CYP2D6* genetic testing as part of clinical care. Since the observed allele frequencies are similar to those previously published for healthy subjects (Table 3), however, we believe that the frequencies observed are representative of major ethnic groups in the US population. Nonetheless, this study does not capture all ethnic groups in the US (e.g., Native Americans) or immigrants to the US from other regions of the world.

We tested for a discrete set of *CYP2D6* alleles, selected out of over 100 *CYP2D6* alleles that have been identified in major ethnic groups present in the US (Gaedigk et al., 2017). It is likely that some of the patients in our study population may have undetected novel or rarer alleles, including structural variants, which may or may not be functionally characterized. This limitation may impact the observed frequency of *CYP2D6* alleles, especially the wild-type *CYP2D6* ^*^1 allele, in the US population. Full gene or long-range sequence-based approaches may be better able to capture rare alleles (Qiao et al., 2016).

In addition, while our *CYP2D6* assay is able to detect many structural variants including multiplications and tandems, the assay is not able to precisely identify numbers of gene copies >3. This limits the ability of this study to observe copy number variation. Furthermore, as a PCR-based test, results are not phased and therefore errors of phasing could occur. Precise identification of phased copy number is an important subject for future studies to further clarify the contribution of this type of variation to functional outcomes.

Approximately half (54%) of the patients tested did not report ethnicity. Other laboratories have also observed significant numbers of patients without ethnicity information (Yao et al., 2014; Beoris et al., 2016). There may be several reasons why physicians do not enter ethnicity information. Since it is not required, it may be omitted due to time constraints, lack of interest or an unwillingness to assign ethnicity in today's admixed and racially-sensitive US culture. In addition, a substantial proportion of individuals (2.5%) are categorized as belonging to two or more races. Of note, allele frequencies for all patients with ethnicity information closely matched those observed in the “Not Reported” group (data not shown). Furthermore, for most of the alleles, the frequencies for the “Not Reported” group most closely matched the frequencies from the Caucasian group. While this may indicate the absence of other ethnic groups, the high number of Caucasians may mask the contribution of these other groups.

When compared to the US Census, our study also showed underrepresentation of Asians and Hispanics compared to other ethnic groups. Therefore, it is possible the observed allele frequencies in Asians and Hispanics might not be representative of the US population. However, the allele frequencies observed in these ethnic groups were within the range of expected values. Moreover, compared to previously published studies, the number of Asians in this study are comparable and the number of Hispanic individuals is higher (Hicks et al., 2015).

To translate genotype into phenotype, we used the system described by Owen et al. (2009) and referred to by the American College of Medical Genetics and Genomics (Lyon et al., 2012). There are other methods to assign phenotype, such as the activity score system (Gaedigk et al., 2008; Hicks et al., 2013, 2015; Crews et al., 2014). Notably, differences in phenotype assignments affect IMs and NMs. For example, CYP2D6^*^17xN/^*^17xN and ^*^17/^*^17 are predicted IMs in our study, but are classified as NMs using the activity score system (^*^17/^*^17 (activity score = 1) and 17xN/^*^17xN (activity score = 2 assuming *N* = 2); both scores are predicting NM). Per Clinical Pharmacogenetics Implementation Consortium guidelines, selection of a non-opioid for pain relief (Crews et al., 2014) or reduction of dose for tricyclic antidepressants (Hicks et al., 2013) is recommended for patients with an IM phenotype, but no clinical action is recommended for NMs. As such, using the phenotype assignment described in Owen et al. (2009) classifies a larger number of patients as IMs triggering a recommendation for clinical action. There are currently no standards for translating genotype into phenotype (Hicks et al., 2014), although standardized terms for CYP2D6 phenotype have been developed (Bell et al., 2017). There is consensus in the pharmacogenetics community that standards are urgently needed to make phenotype prediction more consistent and transparent, and among laboratories, there is an ongoing effort toward developing such standards (Kalman et al., 2016).

In conclusion, *CYP2D6* allele frequencies were determined for a large US population. The results indicate a substantial contribution of structural variants to CYP2D6 function. Discrepancies between observed and previously published results indicate differences in testing and how alleles are assigned. Our findings support recommendations for the standardization of pharmacogenetics allele nomenclature and testing (Kalman et al., 2016). Standardization would make results more comparable across laboratories, and also simplify result reporting and interpretation for clinical use. Future pharmacokinetic studies to compare the level of activity of variants, including structural variants, would also be critical to enable more precise prediction of CYP2D6 activity.

## Author contributions

AD, AM, and TM designed the study; FE, MD, DR, GZ, JV, AD, and TM developed the clinical genotyping process, including assay development and data analysis algorithms; FE and JV performed the clinical genotyping; AD and AM: analyzed the genotyping data and wrote the manuscript; AM and MD analyzed the demographic and ethnic data; AD, GZ, and TM reviewed and made substantial edits to the manuscript. All authors reviewed and approved the manuscript.

### Conflict of interest statement

All of the authors were employed by Millennium Health, LLC at the time this study was completed.
