# Supplementary material for: Frequency of CYP2D6 Alleles Including Structural Variants in the United States
Source: Front Pharmacol. 2018 Apr 5;9:305. doi: 10.3389/fphar.2018.00305 (PMC5895772; doi:10.3389/fphar.2018.00305)
Supplement: Supplementary file 1 [file Table1.DOCX]

**Supplemental Table S1.** Assignment of alleles based on known defining variant(s) as shown by the Pharmacogene Variation Consortium (PharmVar) at [www.PharmVar.org](http://www.PharmVar.org) (Gaedigk et al. 2017).

| **Allele** | **Variant(s) defining the allele** | |
| --- | --- | --- |
|  | **rsID** | **Nucleotide change(s)** |
| ****2*** | rs16947 | 2850C>T |
| ****2A*** | rs16947 | 2850C>T |
|  | rs1080985 | -1584C>G |
| ****3*** | rs35742686 | 2549delA |
| ****4*** | rs1065852 | 100C>T |
|  | rs3892097 | 1846G>A |
| ****4N*** | rs1065852 | 100C>T |
|  | rs3892097 | 1846G>A |
|  | - | gene conversion in exon 9 |
| ****5*** | - | whole gene deletion |
| ****6*** | rs5030655 | 1707delT |
| ****9*** | rs5030656 | 2615_2617delAAG |
| ****10*** | rs1065852 | 100C>T |
| ****17*** | rs16947 | 2850C>T |
|  | rs28371706 | 1023C>T |
| ****29*** | rs16947 | 2850C>T |
|  | rs59421388 | 3183G>A |
| ****35*** | rs16947 | 2850C>T |
|  | rs1080985 | -1584C>G |
|  | rs769258 | 31G>A |
| ****36*** | rs1065852 | 100C>T |
|  | - | gene conversion in exon 9 |
| ****41*** | rs16947 | 2850C>T |
|  | rs28371725 | 2988G>A |

**Supplemental Table S2.** Example genotype assignments for 2 samples tested on the CYP2D6 assay panel. Detection of allele-defining variants is used together with copy number evaluation to determine genotype.

| **ALLELE-DEFINING VARIANT DETECTION** | | | |
| --- | --- | --- | --- |
| **Variant** | | **Sample 1 results** | **Sample 2 results** |
| -1584C>G | rs1080985 | C/G | C/C |
| 31G>A | rs769258 | G/G | G/G |
| 100C>T | rs1065852 | C/T | C/T |
| 1023C>T | rs28371706 | C/C | C/C |
| 1707delT | rs5030655 | T/T | T/T |
| 1846G>A | rs3892097 | A/G | G/G |
| 2549delA | rs35742686 | A/A | A/A |
| 2615_2617delAAG | rs5030656 | AAG/AAG | AAG/AAG |
| 2850C>T | rs16947 | C/T | C/C |
| 2988G>A | rs28371725 | G/G | G/G |
| 3183G>A | rs59421388 | G/G | G/G |

| **COPY NUMBER EVALUATION** | | | | |
| --- | --- | --- | --- | --- |
| **Applicable assays** | **Sample 1 results** | | **Sample 2 results** | |
| exon 9 | 3 | | 1 | |
| intron 6 | 3 | | 3 | |
|  | Proportion of wild-type | Proportion of variant | Proportion of wild-type | Proportion of variant |
| -1584C>G | 2 | 1 | 3 | 0 |
| 100C>T | 1 | 2 | 1 | 2 |
| 1846G>A | 1 | 2 | 3 | 0 |
|  |  |  |  |  |
| **CYP2D6 GENOTYPE ASSIGNMENT** | | | | |
|  | **Sample 1** | | **Sample 2** | |
|  | *2A/*4xN | | *1/*36xN | |
